# NMR spectroscopy of single sub-nL ova with inductive ultra-compact single-chip probes

**DOI:** 10.1038/srep44670

**Published:** 2017-03-20

**Authors:** Marco Grisi, Franck Vincent, Beatrice Volpe, Roberto Guidetti, Nicola Harris, Armin Beck, Giovanni Boero

**Affiliations:** 1 Microengineering Institute, École Polytechnique Fédérale de Lausanne (EPFL), Lausanne, 1015, Switzerland; 2 Bruker BioSpin AG, Industriestrasse 26, Fällanden, 8116, Switzerland; 3 Global Health Institute, École Polytechnique Fédérale de Lausanne (EPFL), Lausanne, 1015, Switzerland; 4 Department of Life Sciences, University of Modena and Reggio Emilia, Modena, 41125, Italy

## Abstract

Nuclear magnetic resonance (NMR) spectroscopy enables non-invasive chemical studies of intact living matter. However, the use of NMR at the volume scale typical of microorganisms is hindered by sensitivity limitations, and experiments on single intact organisms have so far been limited to entities having volumes larger than 5 nL. Here we show NMR spectroscopy experiments conducted on single intact ova of 0.1 and 0.5 nL (i.e. 10 to 50 times smaller than previously achieved), thereby reaching the relevant volume scale where life development begins for a broad variety of organisms, humans included. Performing experiments with inductive ultra-compact (1 mm^2^) single-chip NMR probes, consisting of a low noise transceiver and a multilayer 150 μm planar microcoil, we demonstrate that the achieved limit of detection (about 5 pmol of ^1^H nuclei) is sufficient to detect endogenous compounds. Our findings suggest that single-chip probes are promising candidates to enable NMR-based study and selection of microscopic entities at biologically relevant volume scales.

Nuclear magnetic resonance (NMR) is a well-established spectroscopic technique widely employed in physics, chemistry, medicine, and biology. It allows for experiments on living matter^1^,^2^, whose relevance in biology is proven by developments such as *in vivo* protein structure determination^3^, metabolic profiling^4^, visualization of gene expression^5^, and latent phenotype characterization^6^. Despite its advantages, NMR suffers from a significantly lower sensitivity with respect to other methods. As a result, experiments are often restricted to large ensembles of cells^1^,^3^,^4^,^6^.

Single cell studies are necessary to investigate heterogeneous phenomena within a cell population^7^,^8^,^9^. Recently, a number of techniques were applied to intracellular metabolic profiling at single cell scale, all having different limitations and degree of invasivity. For instance, mass spectrometry and fluorescence labeling allow high sensitivities, but require cellular content extraction or selective labeling with fluorophores^7^,^9^. Questions concerning invasivity stimulated the coin of the biological equivalent of the so called observer effect, referring to the inability to separate a measurement from its potential influence on the observed cell^9^. In this regard, NMR is one of the most promising techniques for studies of intracellular compounds in untouched living entities (i.e., with extremely weak physical and chemical perturbations)^1^,^7^.

The application of NMR to intact individual microscopic biological entities was previously reported down to a volume of 5 nL. The first single-cell NMR experiments were performed on *Xenopus laevis* ova^10^ which have volumes of about 1 μL. Later, single giant neurons of *Aplysia californica,* with volumes of approximately 10 nL, were studied^11^. The particularly large volumes of these cells allowed several pioneering studies such as the profiling of highly concentrated metabolites and their subcellular localization^12^,^13^, imaging of *Xenopus laevis* cleavage^14^ and neurons structure^15^, and study of water diffusion properties within the cytoplasm and nucleus^10^,^11^,^16^,^17^,^18^. Recently, also spectroscopy of a single adult *C. elegans* worm (about 5 nL volume) was reported^19^.

In this work we report, for the first time, NMR-based spectroscopy of single untouched sub-nL ova, specifically describing experiments on the tardigrade *Richtersius coronifer (Rc*) and the nematode *Heligmosomoides polygyrus bakeri (Hp*). These ova are just two of the many models present at the sub-nL scale (Fig. 1a), which include numerous species of microorganisms, echinoderms, and mammals (humans included)^20^. *Rc* ova are spherical with conical processes on the cuticular surface of the egg shell and have a typical volume of 0.5 nL (Fig. 1b). *Hp* ova are ellipsoidal and have a typical volume of about 0.1 nL (Fig. 1c). NMR spectroscopy of sub-nL biological samples is both a volume and concentration limited problem, setting severe constraints on the required spin sensitivity. Here we employ a recently developed single-chip integrated inductive NMR probe^21^ entirely realized with a commercially accessible complementary-metal-oxide-semiconductor (CMOS) technology, where the combination of a low noise transceiver and a multilayer microcoil allows for high spin sensitivities in sub-nL volumes (Fig. 1d). In brief, the entire NMR probe occupies an area of about 1 mm^2^, it has a sensitive region of about 200 pL (on top of the microcoil) with a spin sensitivity at 7 T of about 1.5 × 10^13^ spins/Hz^1/2^, and its planar geometry allows for a relatively easy access to the sensor. In order to use the device for the spectroscopy of sub-nL ova of microorganisms, we manually place the sample in the sensitive region of the probe using a polystyrene cup filled by agarose gel (see Methods). Figure 1e describes the assembled probe where single ova are in contact with the microcoil surface and embedded in the gel. This setup systematically allows for experimental times as long as one day.

## Results

### Linewidth in *Rc* ova

Figure 2a shows three ^1^H NMR spectra obtained at 7 T (300 MHz) from single *Rc* ova embedded in H_2_O-based agarose gels. Due to a measured linewidth of about 70 Hz, the strong water signal (used as internal chemical shift reference at 4.7 ppm^17^) overlaps with nearby resonance lines. The relatively short spin-spin relaxation times typically observed in oocytes explain only partially these broad lines^13^,^17^,^18^ that must be caused by susceptibility mismatches. In order to investigate the origin of the field distortions we performed measurements with an alternative setup enabling the spectroscopy of these samples in pure water and with controlled and reduced hardware-related field distortions (see S.I). Repeated experiments suggest that the linewidth measured in *Rc* ova is intrinsically related to the sample, probably resulting from microscopic constituents of the ovum introducing susceptibility mismatches whose typical spatial distribution impedes field shimming in the intracellular region. In line with this observation, previous studies limited to the vegetal cytoplasm of intact *Xenopus laevis* ova attributed similarly broad linewidths (about 0.3 ppm) to the presence of yolk platelets, or other organelles with paramagnetic components, generating local susceptibility mismatches^13^. However, despite the relatively low spectral resolution that characterizes *Rc* ova, the achieved limit of detection (advantageous in the setup employing the integrated single-chip probe) is sufficient for a qualitative detection of intracellular compounds (Fig. 2a).

### *Rc* ova spectroscopy

In presence of susceptibility mismatches enlarging the water signal it is difficult to apply water suppression techniques without introducing significant spectral artifacts^22^. As an alternative to the use of water suppression techniques we embedded the biological sample in gels based on heavy water (D_2_O), thus eliminating the water signal by replacement of water with D_2_O. In D_2_O-based agarose gels, HDO is formed by proton exchange with the OH groups in the agarose molecule. HDO resonates at about 0.03 ppm relative to the H_2_O chemical shift^23^ and contributes to the only background signal that is visible in our experimental conditions and time scales (Fig. S2). The weaker background signal in D_2_O gels (about 100 times smaller than in H_2_O gels) is reproducible, allows one to better resolve the resonance lines close to water, and can be used as internal chemical shift reference (at 4.7 ppm as water). We do not exclude that, in presence of the sample, the peak at 4.7 ppm results also from leftover H_2_O within the ova.

Figure 2b shows NMR spectra of eight single *Rc* ova in D_2_O-based gels obtained by dispersing agarose in pure heavy water. These spectra exhibit linewidths and chemical shifts compatible with the ones observed in H_2_O-based gels. A detailed comparison among spectra of different ova seem to indicate that the *Rc* ova exhibit a certain degree of spectral heterogeneity (Fig. 2c). In what follows we discuss the possible experimental artifacts that could lead to artificial spectral diversities and show a reproducibility study of single ova spectra.

*Rc* ova are randomly selected from a population where there is no control over fertilization and/or development stage. Their volume is approximatively spherical, with a diameter naturally varying from 100 to 130 μm. As shown in detail by the sensitivity maps in Fig. S3, the most sensitive region of our excitation/detection microcoil roughly corresponds to a deformed semi-ellipsoid of about 200 pl, i.e. smaller than the ova volume. Consequently, the signal amplitude does not depend linearly on the ovum volume. In order to quantitatively estimate the dependence of the signal amplitudes on the natural variability of ova volumes, we performed a numerical integration of the effective sensitivity shown in Fig. S3 over spherical volumes (representing *Rc* ova) having diameters of 100 and 130 μm, placed on top of the microcoil, in which an homogeneous spin density is considered. The result of this calculation indicate that the maximum variability of signal amplitude due to different ova volumes is of about 25%. This value slightly increase to about 30% when the smaller sphere is laterally displaced by 15 μm with the respect to center of the microcoil. From this estimation, we deduce that the variability in terms of signal amplitudes shown in Fig. 2 (as large as 350%) cannot be explained by the natural variability of ova volumes and/or the ovum-to-microcoil misalignment.

Other factors that might provoke artificial heterogeneity among these NMR spectra can be: (1) the non-homogeneous coil sensitivity combined with a non-uniform intracellular chemical composition; (2) the random orientation of the ovum within the structural field inhomogeneity of the setup; (3) the presence, upon sample placing, of invisible air bubbles at the microchip-sample-gel interface (see Methods for assembly procedure). In order to investigate these possible sources of artifacts, we performed six additional experiments on four *Rc* ova, in particular on the ova which produced the spectra (d), (e), (f) and (g) shown Fig. 2b. Figure 3a shows spectra of ovum (d) and ovum (e) after three arbitrary repositioning, realized delicately rotating the ova within the respective spent gels. Although we observe some variations of the linewidths as well as of the signal amplitudes, the dominant spectral features (i.e. the ones between 0 and 4 ppm) are conserved upon sample rotation and change of local environment. Figure 3b shows the result of experiments where both ovum (f) and (g) are repositioned in a fresh gel. As we can see, the dominant spectral features were conserved also upon transfer into fresh gels. Figure 3c and d compare the averaged spectra of ova (f) and (g) to spectra of ova (a) and (h), showing that the variability in spectra of different ova can be larger than the variability of repeated experiments on the same ovum. Overall, Fig. 3 suggests that the observed diversity among spectra of *Rc* ova cannot be attributed only to the manipulation and positioning of the ovum but must be caused, at least partially, by its intrinsic properties.

### *Hp* ova spectroscopy

**Figure 1:**
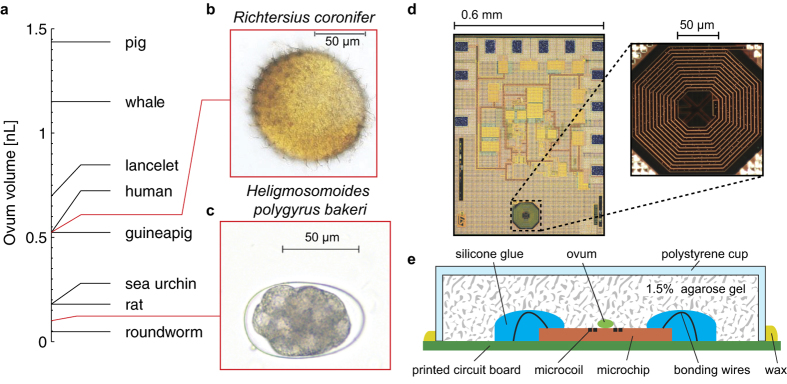
Samples and setup. (**a**) Approximate volumes of ova of selected animals. (**b,c**) Typical bright field images of the ova studied in this work. (**d**) Photographs of the integrated microchip and microcoil (details in Ref. 21). (**e**) Schematic representation of the single ovum probe in section view.

Figure 4a shows ^1^H NMR spectra obtained from three different single *Hp* ova placed in D_2_O-based gel, resulting from 36 hours of averaging and characterized by a linewidth of about 0.25 ppm. *Hp* ova, whose ellipsoidal shape was systematically oriented horizontally on the gel, were always entirely contained within the most sensitive region of the probe (see Figs 1c and S3) allowing for a full exploitation of the microcoil high sensitivity. Contrarily to the case of *Rc* ova, in these measurements on *Hp* ova we do not observe a clear indication of heterogeneity despite the study is performed at comparable effective sensing capability (see below). With a typical volume of about 0.1 nl the *Hp* ovum is, to date, the smallest intact biological sample in which intracellular compounds are detected with NMR spectroscopy. Figure 4b shows averaged NMR spectra obtained from experiments on *Rc* and *Hp* ova in D_2_O-based gels. These spectra indicate that, at our level of sensitivity, the averaged intracellular chemical compositions of *Rc* and *Hp* ova are similar, with only small eventual differences at 3.2 and 3.8 ppm between the two species.

### Sensitivity of the single-chip probe

Due to the planar geometry of the excitation/detection coil, our probe has an effective spin sensitivity which depends on the sample volume, shape, and distance from the coil surface. Our experimental conditions are characterized by a spectral resolution of about 0.3 ppm and a field strength of 7 T. In the case of a spherical sample of 30 μm diameter in contact with the chip surface, the time-domain spin sensitivity of about 1.5 × 10^13^ spins/Hz^1/2^ corresponds to a limit of detection (LOD) in the frequency domain of about 700 pmol of ^1^H nuclei per single scan (quantity of ^1^H nuclei that gives a signal-to-noise ratio of three). In this example the sensing capability of the microcoil is fully exploited as the sample is contained within the most sensitive region of the detector (see S3). In the case of a spherical sample of 100 μm diameter in contact with the chip surface, the spin sensitivity is reduced to about 4 × 10^13^ spins/Hz^1/2^, corresponding to an LOD in the frequency domain of about 1900 pmol of ^1^H nuclei per single scan (the spins intended to be distributed homogeneously within the whole sample). In terms of LOD, the performance of our single-chip probe are competitive with the most sensitive inductive NMR devices so far reported^24^,^25^,^26^,^27^.

### Chemical shifts in *Rc* and *Hp* ova

In this study the relatively small number of samples available (see Methods) poses significant and non-trivial technical challenges to studies of ova collections aimed at the elucidation of proton peaks assignment (see Discussion). In our experiments the peak assignment is hindered by the combination of a small number of spins with a relatively poor spectral resolution. Nevertheless, a few qualitative observations can be done by comparison to previously reported NMR spectra of intact *C. elegans* worms^28^ and *Xenopus laevis* ova^13^,^17^. Although these NMR-based studies analyze biological entities that are different from the ones investigated in this work, they probably represent the closest term of comparison available in literature in terms of volume size and samples nature. The NMR signals in *Xenopus laevis*^13^,^17^ at about 0.9, 1.3, 2.1, 2.8, 5.2 ppm were attributed to highly concentrated yolk lipids (in particular triglycerides^13^,^29^). These results well explain the origin of the dominant features in both *Rc* and *Hp* ova spectra. In Fig. 4a and b, a peak at about 3.2 ppm seems to discriminate the intracellular content of *Hp* ova from the one of *Rc* ova. Prominent resonances at about 3.2 ppm were previously assigned to a relatively restricted group of metabolites in intact *C. elegans* worms, which are nematodes as *Hp*^28^.

**Figure 2:**
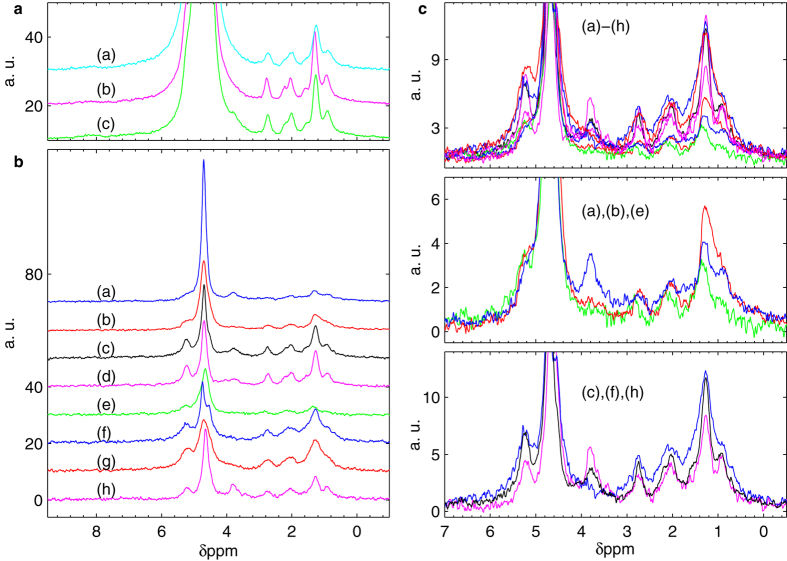
NMR spectroscopy of single *Richtersius coronifer (Rc*) ova. (**a**) Three *Rc* single ovum experiments in H_2_O-based gels realized by dispersing 1.5% agarose in either M9 buffer (a) or pure H_2_O (b–c). (**b**) Eight *Rc* single ovum experiments in D_2_O-based gels realized by dispersing 1.5% agarose in pure D_2_O. (**c**) Detailed comparison of *Rc* ova in D_2_O-based gels. Colors refer to the same spectra as in (**b**).

**Figure 3:**
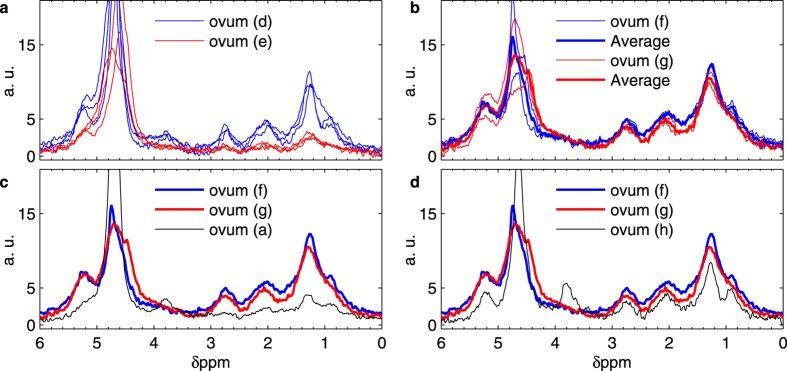
Reproducibility study of *Richtersius coronifer (Rc*) spectra. (**a**) Measurements of two ova, ovum (**d**) and ovum (**e**) as indicated in Fig. 2b. Each ovum was arbitrarily repositioned three times within the respective spent gels. Each spectrum results from 12 hours of averaging. (**b**) Measurements of two ova, ovum (**f**) and ovum (**g**) as indicated in Fig. 2b. Each ovum was arbitrarily repositioned twice within fresh gels. Each spectrum results from 12 hours of averaging. (**c**) Comparison of averaged spectra of ova (**f**) and (**g**) with spectrum of ovum (**a**) as indicated in Fig. 2b. (**d**) Comparison of averaged spectra of ova (**f**) and (**g**) with spectrum of ovum (**h**) as indicated in Fig. 2b.

**Figure 4:**
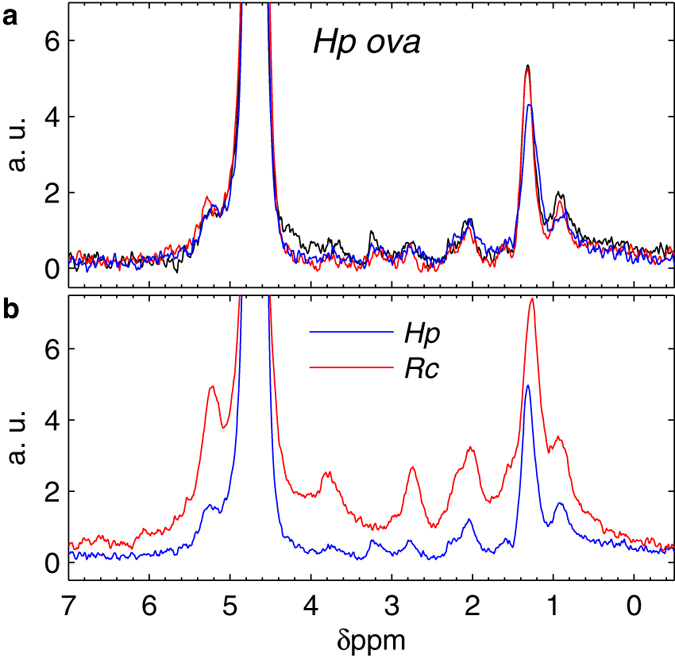
NMR spectra of single *Heligmosomoides polygyrus bakeri* (*Hp*) ova and averaged spectra of *Richtersius coronifer* (*Rc*) and *Hp*. (**a**) Three *Hp* single ovum experiments in D_2_O-based gels. (**b**) Comparison between average spectra of five *Rc* ova (red) and three *Hp* ova (blue) in D_2_O-based gels.

As shown in Fig. 2 a visible signal at 3.8 ppm is present in some *Rc* ova. A resonance at about 4 ppm was assigned to the glycerol backbone in *Xenopus laevis*, typically lower and broader with respect to the other lipid signals (to which this compound is strictly related)^13^. Hence, this resonance is hardly related to yolk lipids. The presence of a highly concentrated endogenous compound is a more likely explanation for the signal detected at this particular chemical shift.

## Discussion

In this study we reported on the use of a state of art sub-nL NMR probe for the analysis of single sub-nL ova of microorganisms, indicating the limits of the technique for the non-invasive detection of intracellular compounds within ova as small as 0.1 nl. The results shown may be used as a starting point to extrapolate the realistic experimental possibilities offered by NMR tools for applications such as the non-invasive selection of microscopic entities based on the direct quantification of highly concentrated endogenous compounds. In terms of spin sensitivity performance that future setups may offer, a straightforward improvement is the use of a higher field. Moving from 7 T to 23.5 T (the highest field commercially available) with the same microcoil should improve the spin sensitivity by a factor of six if the linewidth originates entirely from magnetic susceptibility issues (see S.IV). In these conditions it is reasonable to achieve limits of detection on ^1^H nuclei in the order of 7 pmol in 10 minutes and 0.9 pmol in 10 hours for samples having a volume below 100 pl and linewidths as large as 0.3 ppm. Further improvements are obvious for samples exhibiting typical linewidths narrower than the ones observed in this study.

Improved spectral resolutions may be obtained by MAS techniques^13^,^19^,^30^. A few explorations on microscopic intact biological samples report linewidths of about 0.1 ppm in *Xenopus laevis* eggs at 14 T^13^ and *C. elegans* at 23.5 T^19^. Experiments on large collections of *C. elegans* and bovine tissues demonstrate linewidths as narrow as 0.05 ppm^19^,^30^. It seems therefore reasonable to obtain significant narrowing of the line via MAS. Although MAS probes are not yet optimized for maximum sensitivity at the sub-nL scale, its application at larger volume scales (few tens of nL) may already provide tools supporting the study of sub-nL ova. In wider terms, static and/or spinning probes analyzing 10 nL collections of rare or precious sub-nL ova would allow for proton assignments (elucidating eventual heterogeneities detected among individual samples at the single ovum level) and a better characterization of the spin-spin relaxation properties without need of excessive sample accumulation. However, the realization of such tools is hindered by significant technical challenges, simultaneously requiring small sensitive volumes, high filling factors, high resolution, MAS, and sample loading and manipulation capabilities.

Our results, obtained at a relatively weak field of 7 T, suggest that a LOD of about 5 pmol of ^1^H nuclei within a sub-nL region (in this study specifically corresponding to sensitivities ranging from 20 to 50 mM in terms of intracellular concentration) is sufficient for the detection of the most concentrated compounds in individual ova of microorganisms having volumes below 1 nL. Curiously, signals at chemical shifts that are not typical of yolk lipids are visible. This indication seems, at first sight, in contradiction with the previous NMR spectroscopic studies of intact *Xenopus laevis* ova^13^, where yolk lipids explain all the spectroscopic features, which are essentially identical to those of the yolk of an hen egg^31^. In order to detect metabolites in these samples it was indeed necessary the use of magic angle spinning probes at 14 T loaded with more than one ovum^13^. However, the *Xenopus laevis* ovum (the smallest previously analyzed with NMR spectroscopy) might not be the best term of comparison, as its typical volume (about 1 μL) is larger by a factor ranging from 10^3^ to 10^4^ with respect to the ova studied in this work.

A peculiar class of sub-nL ova that justifies the interest in approaches for the non-invasive intracellular spectroscopy of individual samples is constituted by the mammalian zygotes. Recent studies demonstrate, using techniques other than NMR, that in sheep^32^ and human^33^ oocytes the uptake or production rates of metabolites such as lactate, pyruvate, and glucose can reach 100 pmol/oocyte/h and change radically along the natural development. It is worth noting that these results concern exchange rates measured in the extracellular medium and, hence, do not provide a direct quantification of the intracellular content and its time evolution. Spectrophotometry of intracellular extracts, on the other hand, has shown that up to 30 pmol/oocyte of glutathione (GSH) are contained in oocytes of goat^34^ and pig^35^,^36^ and can change in reaction to environment and developmental stage^37^. Variations of a few pmol/oocyte of GSH in time scales of the order of several hours have been reported in hamster^38^ and rat^39^ oocytes. In these studies, the intracellular GSH content and its evolution is directly measured, but the ensemble measurements hide possible heterogeneities among single entities. These findings indicate that the sensitivity achievable with high sensitivity miniaturized inductive NMR probes should be sufficient for a non-invasive real-time intracellular monitoring of GSH in single mammalian zygotes. The application of NMR spectroscopy to the analysis of spent culture media was recently proposed to aid the selection of viable human embryos for *in vitro* fertilization purposes^40^. The direct application of NMR on single embryos using miniaturized high sensitivity probes is potentially advantageous for this aim. We suggest that systematic and extensive NMR studies on single cultured ova may provide new data that could shed light on cryptic processes involved in embryonic development^32^,^33^,^34^,^35^,^36^,^37^,^38^,^39^ and provide new methodologies to estimate embryonic health^37^,^40^.

The hardware used in this work is an ultra-compact integrated probe entirely realized with commercially accessible complementary-metal-oxide-semiconductor (CMOS) technologies that might open to the realistic possibility of implementing relatively low-cost arrayed miniaturized probes. However, improvements for what concern samples manipulation are required, especially for applications aiming at studying precious samples such as mammalian embryos. Recently, many efforts were successfully dedicated to the microfabrication of devices for manipulation and culture of individual living embryos^41^,^42^. Both integrated circuits and microfluidics are suitable for arrays implementation, and their combination has been demonstrated in applications such as single cell magnetic manipulation^43^ and flow cytometry^44^. We believe that this combination can be extended to NMR applications for the realization of arrayed high sensitivity NMR probes, enabling simultaneous studies on a large number of single biological entities in the same magnet.

## Methods

Experiments and protocols were approved by the SV-biosecurity unit committee of the École Polytechnique Fédérale de Lausanne and carried out in accordance with the experimentation guidelines of the institution.

### Single ovum probe mounting

The ova were first transferred, using a 100 μl pipette, from the tube into a Petri dish filled by 1.5% H_2_O-based agarose gel. Often more than one ovum was found on the Petri dish, in which case the additional samples were left isolated on the gel for eventual later use and stored at 4 °C between successive experiments. Single ova were transferred into a 1.5% agarose gel-filled polystyrene cup using two eyelashes. No visible damage to the ova was provoked during this procedure. The concentration of agarose was carefully chosen, based on repeated assemblies of ova, such that the resulting gel was hard enough to allow a stable placement of the ovum but still sufficiently soft to avoid ovum rupture during the setup assembly (typically happening for gels with more than 3% of agarose). The gel matrix was providing a deformable soft surface to embed and hold the ovum. When placed on the gel, the ovum was protruding from the surface by about half of its volume, hence ensuring an initial physical contact between the ovum and the surface of the microcoil upon placing. Later, the cylindrical polystyrene cup, containing the gel with the ovum on its surface, was positioned on top of the microchip in such a way that the ovum was precisely placed over the microcoil. The local depletion of any visible air bubble was relatively easy and reproducible. The cup was fixed to the printed circuit board with candle wax. The gel keeps the sample in close contact with the coil for days without physically damaging it, whereas the wax prevents gel drying. The microchip was wire bonded to a printed circuit board, with bonding wires electrically isolated by a silicone glue. Figure 1e describes the assembled probe.

### Tardigrade Richtersius coronifer (*Rc*)

Eggs of *Rc* were extracted from a moss sample collected in Öland (Sweden) by washing the substrate, previously submerged in water for 30 min, on sieves under tap water and then individually picking up eggs with a glass pipette under a dissecting microscope. The eggs were shipped within 24 hours in sealed tubes with water and subsequently stored at −20 °C before use. The embryonic development of *Rc* ova is relatively slow, with the eggs hatching in more than 50 days^45^. All experiments were carried out within a week after tube opening. The tube was stored at 4 °C between separated experiments. The NMR experiments were performed in H_2_O, M9, and D_2_O. It is known that prolonged exposure to a high concentration of D_2_O affects living organisms to different extents, from lethal to marginal^46^,^47^. In order to test the effects of D_2_O exposure on *Rc* specimens, 16 eggs and 10 animals were submerged in D_2_O (at 15 °C) for 36 and 24 hours respectively and then transferred in H_2_O. A control group of 16 eggs was kept in H_2_O. The effects of the exposition to D_2_O on the survival of the specimens were not negligible but definitively not systematically lethal: all the animals survived, and a hatching of 84% in the control group and of 63% in those exposed to D_2_O was observed after a time of approximately 2 months. The total amount of *Rc* ova available for this study was of about 120 units.

### Nematode Heligmosomoides polygyrus bakeri (*Hp*)

Eggs of *Hp* were collected from faeces of infected mice. Faeces were first dissolved in water and then washed with a saturated NaCl solution. Floating eggs were collected from the top layer of the solution and washed twice. Final centrifugation in water for 5 minutes at 2000 rpm sedimented clean eggs at the bottom of the tube. The amount of ova typically available at each extraction varied from tens to a few hundred depending on the host organism response to the infection. Fecundated ova of *Hp* develop into a fully embryonated state within 24 hours and within two days stage 1 larvae begin to emerge^48^. In H_2_O-based gels *Hp* ova regularly hatched after a few hours, the emerging larvae migrating far from the sensitive region of the microcoil. In D_2_O-based gels the ova never hatched within two days of observation, hence allowing for the necessary long averaging time. All experiments were carried out within two days after sample extraction. The tube was stored at 4 °C between separated experiments.

### NMR experimental details

NMR experiments were performed in the 54 mm room temperature bore of a Bruker 7.05 T (300 MHz) superconducting magnet. The electronic setup was identical to the one described in details in ref. 21. All experiments employing the single-chip probe were performed with a repetition time of 2 s, a π/2 pulse length of 2.5 μs, and an acquisition time of 400 ms. The time domain data were post-processed by applying an exponential filter with decay of 50 ms. The alphabetic order in Fig. 2b corresponds to the chronologic order of the measurements.

### Chemicals

H_2_O (Sigma Aldrich, 270733). D_2_O (Acros Organics, 166301000). Agarose (BioConcept, Standard Agarose LE-7-01P02-R). Silicone glue (Momentive, RTV118). Polystyrene cup (Semadeni, 10 mm diameter, 5 mm height). The M9 buffer is prepared as in ref. 49.

## Additional Information

**How to cite this article**: Grisi, M. *et al*. NMR spectroscopy of single sub-nL ova with inductive ultra-compact single-chip probes. *Sci. Rep.* **7**, 44670; doi: 10.1038/srep44670 (2017).

**Publisher's note:** Springer Nature remains neutral with regard to jurisdictional claims in published maps and institutional affiliations.

## Supplementary Material

Supplementary Information
