# Supplementary material for: NMR spectroscopy of single sub-nL ova with inductive ultra-compact single-chip probes
Source: Sci Rep. 2017 Mar 20;7:44670. doi: 10.1038/srep44670 (PMC5357791; doi:10.1038/srep44670)
Supplement: Supplementary Information [file srep44670-s1.pdf]

Supplementary Information

NMR spectroscopy of single sub-nL ova with inductive ultra-compact single-chip probes

Marco Grisi*, Franck Vincent, Beatrice Volpe, Roberto Guidetti, Nicola Harris, Armin Beck, Giovanni Boero

S.I: Additional experiments on *Rc* ova

Micro-fabricated microcoils whose copper loops are fully embedded in SU-8 photo epoxy (Bruker Biospin) probably represent the best commercially available alternative to CMOS microcoils. Among the products offered, we selected planar microcoils characterized by 5 copper loops, internal diameter of 50 µm and external diameter of 250 µm. The resonating microcoil, fine-tuned with variable capacitors (2322-1G, Johanson), is connected to a single chip transceiver identical to the one used in the fully integrated probe. On top of the microcoil a few microns thick bio-compatible layer acts as protective layer and working surface. In order to analyze liquid samples and single ova, 150 µm diameter PET capillaries having 6 µm thick walls (Vention Medical) are placed on the top of the microcoil in the same direction as the vertical static magnetic field. This particular configuration allows for measured linewidths as narrow as 3 Hz in pure water. Figure S1 shows the typical spectra of the liquid within the capillary at a distance of about 1 mm from the ovum and at the ovum position, both after shimming of the magnetic field. The room temperature shim system of our magnet, whose maximum gradient corresponds to about 0.4 Hz/µm, resulted very effective when the sensitive region was occupied by the liquid sample. On the contrary, when the ovum was aligned to the microcoil, lineshape and linewidth of the water signal remained substantially unchanged for any setting of the shimming system. The measured linewidth at the ovum position is about 0.3 ppm, i.e. about a factor 30 larger than the water peak measured at 1 mm distance from the ovum within the same capillary. This experience, repeated five times, suggests that the linewidth measured in *Rc* ova results from mismatches localized within the sample, whose typical spatial distribution impedes field shimming in the sample region fixing lineshape and linewidth independently from the applied gradients.

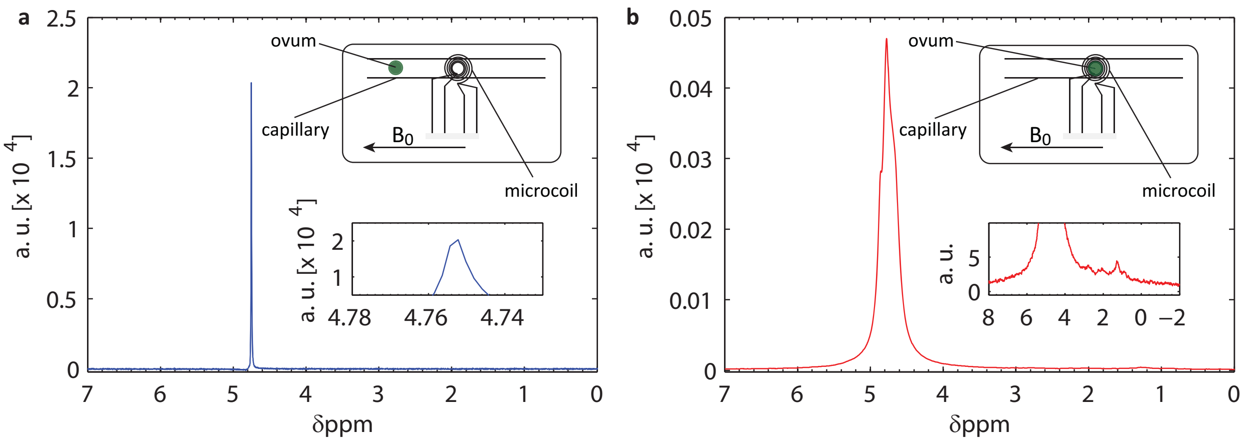

Fig. S1. Additional experiments of single *Richtersius coronifer* (*Rc*) ova in pure H_2O. (**a**) Spectrum of about 800 pl of H_2O at about 1 mm from the ovum position resulting from 30 averaged scans. The acquisition time is 1.6 s, and no filters are applied on the time domain signal. The resulting linewidth is about 0.01 ppm, i.e. about 3 Hz (see inset). (**b**) Spectrum at the ovum position resulting from 18000 averaged scans. The acquisition time is 0.8 s. The resulting linewidth is about 0.3 ppm, i.e. about 90 Hz. Details of the single ovum spectrum are shown in the inset (the time domain data were post-processed by applying an exponential filter with decay of 50 ms in order to improve the signal-to-noise). Both spectra in (**a**) and (**b**) are acquired at the best shimmed magnetic field. The experiments are performed with a repetition time of 2 s and a pulse length of 2.5 µs (maximizing the signal).

S.II: Background characterization

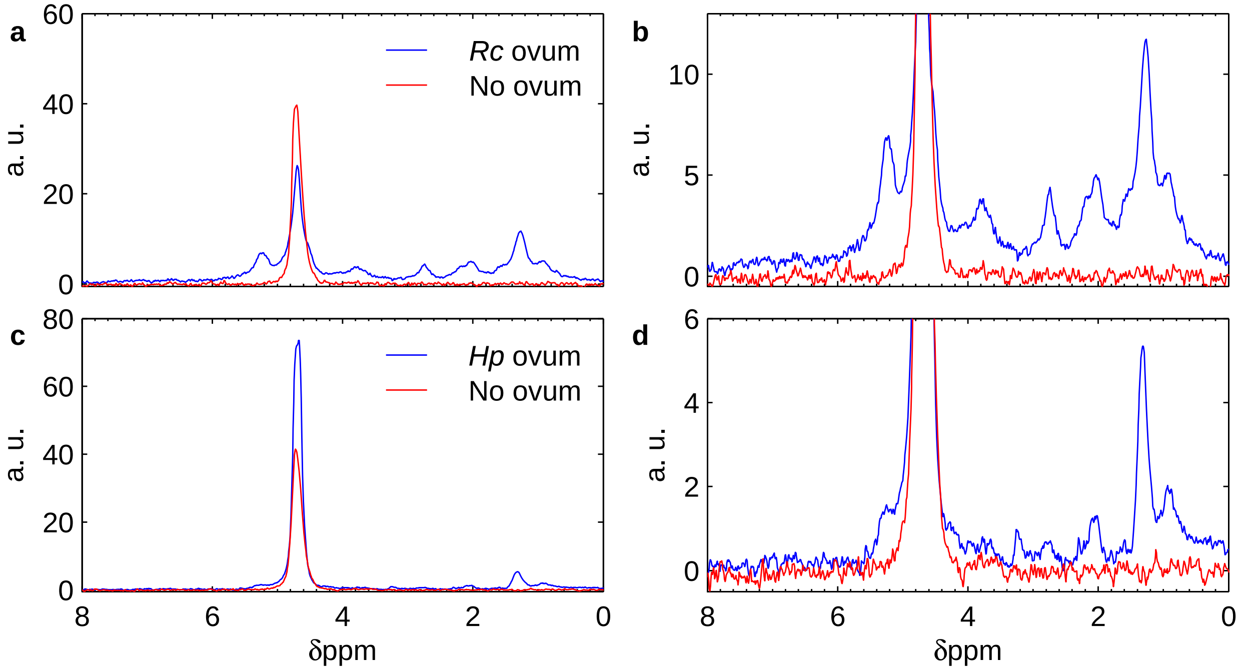

Fig. S2. Comparison between single ova experiments in D_2O-based gel and D_2O-based gel background. (**a**) Spectra of a single *Rc* ovum in D_2O-based gel and of a D_2O-based gel. (**b**) Detailed comparison. (**c**) Spectra of a single *Hp* ovum in D_2O-based gel and of a D_2O-based gel. (**d**) Detailed comparison.

S.III: Microcoil sensitivity map

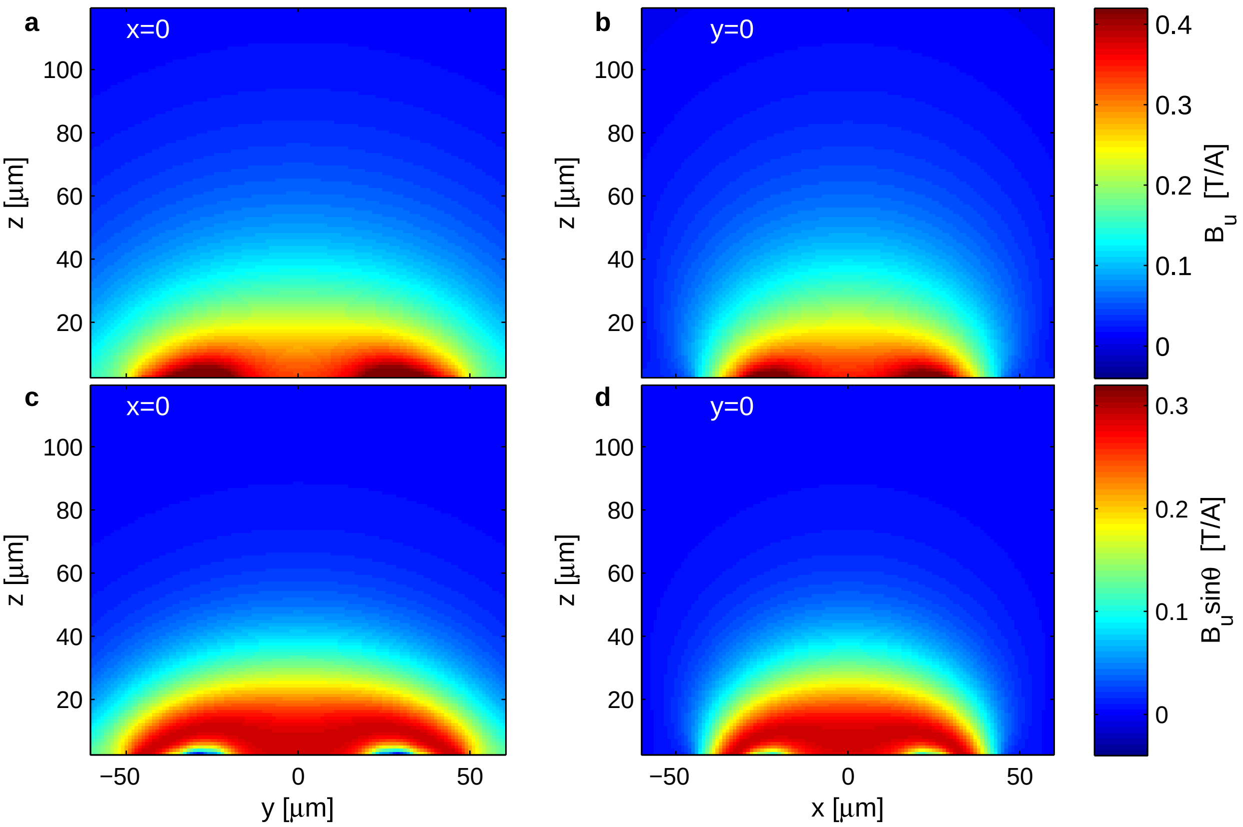

Fig. S3. Maps of effective unitary field and effective sensitivity of the integrated microcoil. The static B_0 field is oriented along the $\overline{x}$ axis. The octagonal loops of the microcoil are approximated with circular ones. The detailed description of the microcoil structure is available in Ref. 21. The unitary field, defined as the field produced by a current of 1 A in the coil loops, is computed via the Biot-Savart law. The effective unitary field B_u is the component orthogonal to the static field. The effective coil sensitivity is computed taking into account the effect of the non-homogeneity of the nutation angle $\theta=\gamma B_1\tau$. The resulting effective sensitivity is $B_u\sin\theta$. The pulse length that maximizes the signal (τ=2.5 μs), is measured experimentally and is matched by simulations that assume a current i=9 mA through the coil in TX mode. (**a-b**) Maps of the effective unitary field $B_u = (B^2_{u,z}+B^2_{u,y})^{1/2}$ at x=0 cross-section (**a**) and y=0 cross-section (**b**). (**c-d**) Maps of the effective coil sensitivity in conditions of maximum total signal (τ=2.5 us) at x=0 cross-section (**c**) and y=0 cross-section (**d**).

S.IV: Sensitivity at higher fields

The free induction decay (FID) NMR signal consists of several contributions at slightly different frequencies. Each contribution has an angular frequency $\omega_0 = \gamma_{eff} B_0$, where γ_{eff} is the effective gyromagnetic ratio of the probed nuclei and depends on their chemical environment. In general, the signal contribution at each frequency is $s(t)=s_0\cos(\omega_0)\exp(-t/T_2^*)$, where T_2^* is the effective decay time depending on the spin-spin relaxation time and the field inhomogeneity in the sample volume, $s_0 = \gamma_{eff}\chi_0 B_0^2 V_s B_u/\mu_0$, χ_0 is the static magnetic susceptibility of the probed nuclei, μ_0 is the vacuum permeability, V_s is the sample volume, B_u is the magnetic field generated by a current of 1 A in the detection coil (i.e., the so called unitary field) [1]. The signal-to-noise ratio (*SNR*) for a single scan is maximized by multiplying the time-domain signal by an exponential function whose decays matches T_2^*. After this operation and for an acquisition time $T >> T_2^*$, the *SNR* in the frequency domain is

$$SNR = \frac{s_0}{\bar{n}}\sqrt{\frac{T_2^*}{2}}\left(1-\exp\left(-\frac{2T}{T_2^*}\right)\right) \simeq \frac{s_0}{\bar{n}}\sqrt{\frac{T_2^*}{2}}$$

where $\bar{n}$ is the noise spectral density [2]. Assuming that the measured linewidths are due to field distortions caused by susceptibility mismatches, the linewidth increases linearly with the static field (i.e., $T_2^* \propto 1/B_0$). Since, as shown above, the signal s_0 scales quadratically with the static field (i.e., $s_0 \propto B_0^2$), the *SNR* scales as $B_0^{3/2}$. Hence, if the field changes from 7 T to 23.5 T, we expect a *SNR* improvement of about six. In this extrapolation of the sensitivity at higher fields, we assumed that the noise spectral density does not depend on the operating field and that the same coil (i.e., same B_u) can be used. This is a good assumption for microcoils having a self-resonance frequency above the operating frequency and a coil metal thickness similar or smaller than the skin depth, as in the case of the integrated microcoil used in this work.

1 Abragam, A. *The Principles of Nuclear Magnetic Resonance*. (Clarendon Press Oxford, 1961).
2 Spencer, R. G. Equivalence of the Time-Domain Matched Filter and the Spectral-Domain Matched Filter in One-Dimensional NMR Spectroscopy. *Concept Magn Reson A* **36A**, 255-265 (2010).
